# Supplementary material for: HIVprotI: an integrated web based platform for prediction and design of HIV proteins inhibitors
Source: J Cheminform. 2018 Mar 9;10:12. doi: 10.1186/s13321-018-0266-y (PMC5845081; doi:10.1186/s13321-018-0266-y)
Supplement: Supplementary file 2 — Additional file 2. Source code of HIVProtI web server. [file 13321_2018_266_MOESM2_ESM.zip › HIVprotI_Source-code/hivprotI/weka-3-6-6/doc/weka/gui/boundaryvisualizer/KDDataGenerator.html]

KDDataGenerator


---


|  |  |  |
| --- | --- | --- |
| **Overview** \| **Package** \| **Class** \| **Tree** \| **Deprecated** \| **Index** \| **Help** \| **Weka's home** | |  |
| **PREV CLASS**   **NEXT CLASS** | **FRAMES**    **NO FRAMES**     **All Classes** |  |
| SUMMARY: NESTED \| FIELD \| CONSTR \| METHOD | DETAIL: FIELD \| CONSTR \| METHOD |  |


---


## weka.gui.boundaryvisualizer Class KDDataGenerator

```
java.lang.Object
  weka.gui.boundaryvisualizer.KDDataGenerator
```

**All Implemented Interfaces:**: java.io.Serializable, DataGenerator

---

``` public class KDDataGenerator extends java.lang.Object implements DataGenerator, java.io.Serializable ```

KDDataGenerator. Class that uses kernels to generate new random
instances based on a supplied set of instances.

**Since:**
:   1.0

**Version:**
:   $Revision: 7059 $

**Author:**
:   Mark Hall

**See Also:**: `DataGenerator`, `Serializable`, Serialized Form

---

**Constructor Summary**

`KDDataGenerator()`


| **Method Summary** | |
| --- | --- |
| `void` | `buildGenerator(Instances inputInstances)`             Initialize the generator using the supplied instances |
| `double[][]` | `generateInstances(int[] indices)`             Generates a new instance using one kernel estimator. |
| `int` | `getKernelBandwidth()`             Get the kernel bandwidth |
| `int` | `getNumGeneratingModels()`             Return the number of kernels (there is one per training instance) |
| `double[]` | `getWeights()`             Get weights |
| `void` | `setKernelBandwidth(int kb)`             Set the kernel bandwidth (number of nearest neighbours to cover) |
| `void` | `setSeed(int seed)`             Initializes a new random number generator using the supplied seed. |
| `void` | `setWeightingDimensions(boolean[] dims)`             Set which dimensions to use when computing a weight for the next instance to generate |
| `void` | `setWeightingValues(double[] vals)`             Set the values for the weighting dimensions to be used when computing the weight for the next instance to be generated |

**Methods inherited from class java.lang.Object**

`equals, getClass, hashCode, notify, notifyAll, toString, wait, wait, wait`

**Constructor Detail**

### KDDataGenerator

```
public KDDataGenerator()
```


**Method Detail**

### buildGenerator

```
public void buildGenerator(Instances inputInstances)
                    throws java.lang.Exception
```

:   Initialize the generator using the supplied instances

    :   **Specified by:**: `buildGenerator` in interface `DataGenerator`
    :   **Parameters:**: `inputInstances` - the instances to use as the basis of the kernels **Throws:**: `java.lang.Exception` - if an error occurs

---


### getWeights

```
public double[] getWeights()
```

:   **Description copied from interface: `DataGenerator`**
:   Get weights

    :   **Specified by:**: `getWeights` in interface `DataGenerator`

---


### generateInstances

```
public double[][] generateInstances(int[] indices)
                             throws java.lang.Exception
```

:   Generates a new instance using one kernel estimator. Each successive
    call to this method incremets the index of the kernel to use.

    :   **Specified by:**: `generateInstances` in interface `DataGenerator`
    :   **Returns:**: the new random instance **Throws:**: `java.lang.Exception` - if an error occurs

---


### setWeightingDimensions

```
public void setWeightingDimensions(boolean[] dims)
```

:   Set which dimensions to use when computing a weight for the next
    instance to generate

    :   **Specified by:**: `setWeightingDimensions` in interface `DataGenerator`
    :   **Parameters:**: `dims` - an array of booleans indicating which dimensions to use

---


### setWeightingValues

```
public void setWeightingValues(double[] vals)
```

:   Set the values for the weighting dimensions to be used when computing
    the weight for the next instance to be generated

    :   **Specified by:**: `setWeightingValues` in interface `DataGenerator`
    :   **Parameters:**: `vals` - an array of doubles containing the values of the weighting dimensions (corresponding to the entries that are set to true throw setWeightingDimensions)

---


### getNumGeneratingModels

```
public int getNumGeneratingModels()
```

:   Return the number of kernels (there is one per training instance)

    :   **Specified by:**: `getNumGeneratingModels` in interface `DataGenerator`
    :   **Returns:**: the number of kernels

---


### setKernelBandwidth

```
public void setKernelBandwidth(int kb)
```

:   Set the kernel bandwidth (number of nearest neighbours to cover)

    :   **Parameters:**: `kb` - an `int` value

---


### getKernelBandwidth

```
public int getKernelBandwidth()
```

:   Get the kernel bandwidth

    :   **Returns:**: an `int` value

---


### setSeed

```
public void setSeed(int seed)
```

:   Initializes a new random number generator using the
    supplied seed.

    :   **Specified by:**: `setSeed` in interface `DataGenerator`
    :   **Parameters:**: `seed` - an `int` value


---


|  |  |  |
| --- | --- | --- |
| **Overview** \| **Package** \| **Class** \| **Tree** \| **Deprecated** \| **Index** \| **Help** \| **Weka's home** | |  |
| **PREV CLASS**   **NEXT CLASS** | **FRAMES**    **NO FRAMES**     **All Classes** |  |
| SUMMARY: NESTED \| FIELD \| CONSTR \| METHOD | DETAIL: FIELD \| CONSTR \| METHOD |  |


---
